# Distribution of *CYP2C19* polymorphisms in Mongolian and Han nationals and the choice of specific antiplatelet drugs

**DOI:** 10.1007/s11096-017-0451-5

**Published:** 2017-06-09

**Authors:** Jing Li, YueXi Wang, HuPing Wang

**Affiliations:** Department of Cardiology, Affiliated Hospital of Inner Mongolia Medical University, Tongdao Road No. 1, Hohhot, 010059 China

**Keywords:** Acute coronary syndrome, Clopidogrel, *CYP2C19* polymorphisms, Mongolia, Pharmacogenetics, Ticagrelor

## Abstract

*Background* Individualized medication reviews may improve our understanding of the distribution of *CYP2C19* polymorphisms in ethnic populations. *Objective* To evaluate differences in *CYP2C19* gene polymorphisms between Mongolian and Han nationals and determine the effect of adjustments of antiplatelet treatments according to the genetic profile in patients undergoing percutaneous coronary intervention (PCI). *Setting* Prospective, observational, single-center study. *Methods* 397 patients diagnosed with acute coronary syndrome were enrolled. Additionally, 186 patients undergoing PCI were given different treatments according to their *CYP2C19* genotypes. Patients with the genotype of an extensive metabolizers (EMs; *1/*1) were co-administered aspirin 100 mg/day and clopidogrel 75 mg/day, following a loading dose of 300 mg; intermediate metabolizers (IMs; e.g., *1/*2 and *1/*3) and poor metabolizers (PMs; e.g., *2/*2 and *2/*3) were administered a loading dose of 180 mg ticagrelor, followed by a maintenance dose of 90 mg twice a day. *Results* In Mongolians, 60.79% of patients were EMs, which was significantly higher than that in Han nationals (*P* = 0.002). In Han individuals, 62.14% of patients were IMs and PMs, which was significantly higher than that in Mongolians (*P* < 0.05). Three patients died, and the frequency of adverse events during follow-up was significantly higher in patients given conventional treatment than in patients given tailored treatment (*P* = 0.039). However, differences in metabolism subtypes did not affect the incidence of adverse reactions. *Conclusions* There were differences in *CYP2C19* polymorphisms between Mongolians and Hans. Effective, safe therapy was achieved by tailoring antiplatelet drug therapy based on genotype.

## Impacts on practice

- *CYP2C19* polymorphisms should be used as a marker for antiplatelet treatment in patients with acute coronary syndrome for the Inner Mongolia region.
- *CYP2C19* testing can guide and provide appropriate antiplatelet therapeutic schedules and prognosis assessments.

## Introduction

As a major metabolizing enzyme, *CYP2C19* is involved in catalyzing the bioactivation of many commonly used drugs in humans [1]. Clopidogrel is a thienopyridine derivative that has antiplatelet effects through the inhibition of platelet adenosine receptors (e.g., P2Y12) after bioactivation by the cytochrome P450 metabolic system [2, 3]. Genetic factors have been extensively studied in clopidogrel-treated patients with acute coronary syndrome (ACS) [4–6]. *CYP2C19* loss-of-function alleles increase the risk of adverse cardiovascular events in patients undergoing percutaneous coronary intervention (PCI) [7]. Several large cohort studies have reported that the diversification of the clopidogrel reaction may be associated with adverse cardiac events; this is especially challenging because clopidogrel plus aspirin is the most frequently prescribed dual antiplatelet therapy [8, 9].

After stent implantation, many patients exhibit stent thrombosis, restenosis, sudden death, and other adverse events, despite taking medications [10]. Such adverse events may be related to the *CYP2C19* genotype. The wild-type CYP2C19*1 allele is associated with normal function of *CYP2C19*-mediated metabolism. The *2 and *3 variants may be considered loss of function genotypes, leading to decreased activation of clopidogrel. The risk of ischemic events is increased with these genotypes, compared to the EMs genotype [11, 12]. Moreover, *CYP2C19* polymorphisms are related to clopidogrel metabolism and affect the efficacy and pharmacodynamic responses in various ethnic and racial groups [13, 14]. Poor metabolizers (PMs) are more frequently of Asian descent than of European descent [7, 15]. Some Chinese studies have shown that clinical effects are associated with *CYP2C19* in Uighurs, Hui, Dai and Tibetan individuals et al. However, no studies have reported *CYP2C19* gene polymorphisms in Mongolians.

In regions such as the Huhhot region in inner Mongolia, with a mixed population of people from Mongolian and Han descent, it is necessary to analyze the differences in *CYP2C19* genotypes in Mongolian and Han individuals. Further studies among subtypes of intermediate metabolizers (IMs) and poor metabolizers (PMs) are also needed to determine the effects of genotype on cardiac adverse events, particularly in patients with ACS taking clopidogrel.

## Aim of the study

Therefore, in this study, we investigated whether there were differences in *CYP2C19* polymorphisms between Mongolian and Han nationals. Furthermore, we assessed the correlation between the 3-month incidence of adverse cardiovascular events and drug effects among different genotypes and subtypes.

## Ethics approval

The protocol of this study was reviewed and approved by the Inner Mongolia Medical University Ethics Committee, and written informed consent was provided by all patients.

## Methods

### Study setting and design

This study was a prospective, observational, single-center study in Inner Mongolia. Main outcome measure is requirement for clinical treatment guidance based on genotype. From February 2015 to March 2016, 397 patients (51 Mongolian, 346 Han) from the Huhhot region, who were all without a history of mixed marriage for three generations, were screened for inclusion in this study. General clinical data, risk factors, and genotype were evaluated. Participants were followed up for morbidity and mortality for 3 months. All participants were 40–70 years of age at baseline.

### Study population

197 patients with ACS who underwent percutaneous coronary intervention (PCI) were studied.

The clinical diagnosis of ACS was based on the 2007 ACC/AHA guideline, including the diagnosis and treatment of acute ST-segment elevation myocardial infarction (STEMI), acute non-ST-segment elevation myocardial infarction (NSTEMI), and unstable angina (UA). Exclusion criteria included contraindications to clopidogrel and aspirin, severe heart failure or left ventricular ejection fraction (%) less than or equal to 30%, bleeding tendency, extremely abnormal liver or kidney function, serious infection, systemic immune diseases, hematologic diseases, cancer, or use of a glycoprotein IIb/IIIa antagonist. Risk factors and medical histories of the patients were recorded.

### Analysis of clinical parameters and genotypes

Records of clinical indicators, including total cholesterol (TC), low-density lipoprotein cholesterol (LDL-c), high-density lipoprotein cholesterol (HDL-c), and history of hypertension, smoking, and drinking, were evaluated in Mongolian and Han individuals. Peripheral blood was obtained from patients (including patients with coronary heart disease and controls) and subjected to gene chip analysis for determination of metabolic phenotype. EMs were considered to have the homozygous wild-type phenotype (genotypes 1*/1*), IMs were considered to have heterozygous mutations (genotypes *1/*2, *1/*3, and *2/*3), and PMs were considered to have homozygous mutations (genotypes *2/*2 and *3/*3) [16]. Analysis of the genotype frequencies of CYP2C19*1, CYP2C19*2, and CYP2C19*3 was performed for Mongolian and Han individuals. Patients with coronary heart disease taking different antiplatelet treatments were divided according to genotype into the conventional treatment group and tailored group. Extensive metabolizers (EMs) were typically given a loading dose of 300 mg clopidogrel and aspirin before undergoing coronary angiography, which was followed by 75 mg clopidogrel daily and 100 mg aspirin. In the tailored group, IMs and PMs received a loading dose of 180 mg ticagrelor and 300 mg aspirin, followed by a maintenance dose of 90 mg twice a day and 100 mg aspirin for 1 year. In addition, patients were also administered other treatments as needed, including β-blockers, angiotensin converting enzyme inhibitors, angiotensin receptor blockers, calcium channel blockers, or statins. Patients were followed up for 3 months, and the relationships between clinical events and genotypes were evaluated. In addition, the demographic information for selected patients, including age, sex, and history of smoking, drinking, hypertension, and diabetes, was collected.

### Follow-up analysis and patient outcomes

**Fig. 1:**
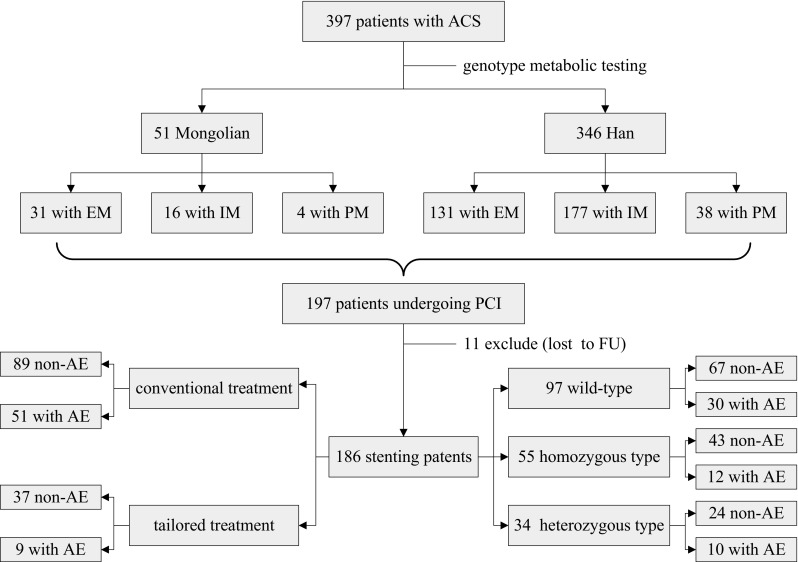
Flow chart of study participants from enrollment to follow-up. *ACS* acute coronary syndrome, *PCI* percutaneous coronary intervention, *FU* follow-up, *EM* extensive metabolizers, *IM* intermediate metabolizers, *PM* poor metabolizers, *AE* adverse events

Eleven patients failed to attend the follow-up visit. Among these patients, three died from cardiac death. Thus, 186 patients completed the follow-up study. One patient developed serious angina pectoris, which was confirmed by angiography for intravascular thrombosis on the second day after operation. Subsequently, the patient underwent intravascular thrombolysis immediately and was given triple antiplatelet therapy, including aspirin, ticagrelor, and warfarin for 3 months, followed by combined treatment with aspirin and ticagrelor. The patient with genotype CYP2C19*3/*3 was in good condition and exhibited stable angina (Fig. 1).

### Detection of *CYP2C19* genetic polymorphisms

Two milliliters of peripheral venous blood was collected from each patient into EDTA-containing tubes in the morning after fasting. We used a *CYP2C19* gene detection kit (Shanghai Bio Technology Co., Ltd., China). All genotyping was carried out using a real-time polymerase chain reaction (PCR)-based ligase detection reaction (Perkin-Elmer Gene Amp PCR Systems 9600; Perkin-Elmer, Shanghai, China). Primers were synthesized by Shanghai Baio Biological Engineering Ltd. (Shanghai, China). Gene chips were placed into a BaioRBE-2.0 Gene Chip Reader (Shanghai Baio Co.) after using a BaioR-Hyb automated hybridization instrument and were subsequently analyzed with BaioRBE-2.0 software for image scanning, data analysis, and output. Experiments were carried out with duplicate samples and negative controls to ensure the accuracy of genotyping.

### Research endpoints

The main endpoints included frequent attacks of angina pectoris within 3 months after PCI, coronary thrombosis, cardiac death, target lesion revascularization, and nonfatal myocardial infarction. Secondary endpoints included unstable angina, nonfatal bleeding, and second stent implantation.

### Statistical analysis

SPSS version 19.0 (SPSS Inc., Chicago, IL, USA) was used to perform all statistical analyses. Data are expressed as the mean ± standard deviation (SD) for continuous variables and numbers and frequencies for categorical variables. Comparisons between groups were made by t-tests for measurement data, whereas comparisons of genotype frequencies between two ethic groups were performed by Chi squared tests. Data conforms to normal distribution and homogeneity of variance. Differences or results with *P* values of less than or equal to 0.05 were considered significant.

## Results

### Patients and baseline characteristics

Demographic characteristics and laboratory data for the study population are summarized in Tables 1 and 2. There were significant differences in TC, LDL-c, HDL-C, and histories of hypertension, smoking, and drinking among Mongolian and Han individuals. In contrast, there were no significant differences in age, sex, triglycerides (TGs), and history of diabetes between Mongolian and Han individuals (*P* < 0.05).

**Table 1:** Demographic and laboratory data in Mongolian and Han individuals

Variables	Mongolian	Han	t	*P*
Age (years)	57.79 ± 13.32	60.17 ± 10.14	1.498	0.182
TC (mg/dL)	4.53 ± 0.82	4.25 ± 0.87	2.154	0.038*
TG (mg/dL)	1.98 ± 0.92	1.91 ± 0.88	0.526	0.599
LDL-c (mg/dL)	2.63 ± 0.74	2.96 ± 0.84	2.661	0. 016*
HDL-c (mg/dL)	0.97 ± 0.17	1.08 ± 0.24	3.142	0.002*

Data are presented as the mean ± standard deviation

*TC* total plasma cholesterol, *TG* triglyceride, *HDL-c*:high-density lipoprotein cholesterol, *LDL-c* low-density lipoprotein cholesterol

* *P* values having statistical significance

**Table 2:** Medical histories in Mongolian and Han individuals

Variables	Mongolian	Han	x^2^	*P*
Sex (male/female)	12/39	107/239	1.158	0.286
Hypertension history (Y/N)	42/9	126/220	38.42	0.000*
History of diabetes (Y/N)	12/39	74/272	0.121	0.731
Smoking history (Y/N)	27/24	91/255	15.102	0.000*
History of drinking (Y/N)	14/37	41/305	9.065	0.002*

Data are presented as percentages

* *P* values having statistical significance

### Metabolic types

Our results showed that the frequency of the extensive metabolizers in Han was significantly lower than in Mongolian individuals, but the frequency of intermediate and poor metabolizers were higher. The metabolic types and frequency distributions are shown in Table 3.

**Table 3:** Distribution of *CYP2C19* metabolic phenotypes in Mongolian and Han individuals

Population	N	EM	IM + PM	x^2^	*P*
Mongolian	51	31 (60.79%)	20 (39.21%)	9.669	0.002*
Han	346	131 (37.86%)	215 (62.14%)		

*EM* extensive metabolizers, *IM* intermediate metabolizers, *PM* poor metabolizers

* *P* values having statistical significance

### Curative effect analysis

Analysis of the curative effects was performed; the frequencies of cardiovascular adverse events upon different treatments are shown in Table 4. In this study more cardiac adverse events occurred in patients with stents who received conventional treatment than in those who received tailored treatment (36.43 vs 19.56%).

**Table 4:** Frequencies of cardiovascular adverse events with different treatments in the follow-up of patients with PCI

Treatment method	N	No. of individuals with AE	No. of individuals with no AE	Frequency (%)	x^2^	*P*
Conventional treatment	140	51	89	36.43	4.319	0.039*
Tailored treatment	46	9	37	19.56		

*PCI* percutaneous coronary intervention, *AE* adverse events

* *P* values having statistical significance

### *CYP2C19* genotypes

There were no significant differences in sub-genotypes in local subjects or cardiovascular adverse events. The results are shown in Table 5.

**Table 5:** Relationship of *CYP2C19* genotype with cardiovascular adverse events in the follow-up of patients with PCI

Genotype	N	No. of individuals with adverse events	No. of individuals with no AE	Frequency (%)	x^2^	*P*
Wild-type	97	30	67	30.93	0.449	0.511
Homozygous type	55	12	43	21.82		
Heterozygous type	34	10	24	29.41		

*PCI* percutaneous coronary intervention, *AE* adverse events

* *P* values having statistical significance

## Discussion

In this study, we investigated the distributions of *CYP2C19* genotypes in Mongolian and Han populations and examined the clinical effects of tailored treatment according to different genotypes in these individuals. We found evidence of significant differences in traditional indicators of cardiovascular diseases, such as TC, LDL-c, HDL-c, and history of hypertension, smoking, and drinking, between Mongolian and Han individuals. Additionally, our results demonstrated that the Mongolian population included more EMs than the Han population (60.79 vs 37.86%, respectively). In Han individuals, IMs and PMs accounted for 62.14% of individuals, which was significantly higher than that in Mongolians. Moreover, conventional treatment and tailored treatment resulted in different clinical outcomes based on genotype in the patients in this study. However, during clinical follow-up, the subtype of abnormal metabolism was not significantly correlated with genotype or cardiovascular adverse events.

*CYP2C19* gene polymorphisms are believed to be important factors affecting clopidogrel drug metabolism. Interindividual variability in platelet reactivity is common among different ethnic groups treated with clopidogrel, and different genetic phenotypes influence the risk of adverse clinical events [14, 17]. However, no studies have shown meaningful effects of ethnicity on clinical outcomes and the clinical effects of individualized drug management, which is therefore not currently warranted [18]. However, in this study, we found *CYP2C19* genetic differences between Mongolian and Han individuals, which may guide clinical decisions in the administration of antiplatelet drugs, thereby affecting clinical outcomes. These findings are consistent with those from previous studies showing that *CYP2C19* mutation analysis should be performed on patients before clopidogrel therapy in order to deter-mine the appropriate choice of antiplatelet drugs [19, 20]. Moreover, many studies have determined the cost effectiveness of genotype testing prior to treatment with common drugs [21].

Several studies have indicated the correlations between different genotypes and cardiac adverse events in clopidogrel-treated patients [22]. However, the explicit outcomes and prognosis associated with *CYP2C19* genotype are unclear. In accordance with some reports [23], our results showed there were no correlations between gene subtype/dysfunction and adverse cardiovascular outcomes. Other genes and environmental factors may also play a significant role, and additional studies are needed to assess these factors. In this study, however, a serious adverse event occurred in a patient with the CYP2C19*3/*3 genotype, which was relatively rare in our population. Thus, more studies are needed to explore the relationship between homozygous PMs and clinical events.

In our study, TC, LDL-c, HDL-c, and history of hypertension, smoking, and drinking were found to be risk factors affecting cardiac adverse events, similar to previous studies. In addition, traditional indicators, including metabolism genotype, still influence the efficacy of drugs. Interindividual heterogeneity in drug responses will facilitate the development of molecular genetic analyses. Indeed, determination of the unique features of patients with ACS may allow tailoring of specific antiplatelet therapies to achieve the maximum cost-benefit ratio. The results of this study provide new insights into genetic polymorphisms in Chinese ethnic minorities and therefore indicate the use of clopidogrel in Inner Mongolia, with potential applications in the development of individualized treatments. We propose that regular doses of clopidogrel are more appropriate for Mongolian individuals, particularly patients in pastoral areas, who exhibit a lower incidence of clopidogrel resistance. In contrast, Han patients are more sensitive to *CYP2C19* genetic polymorphisms, and a tailored treatment is recommended, if possible, in these individuals.

### Study limitations

There were several limitations to this study. First, the study sample was small and biased in terms of subject selection, particularly for the Mongolian cohort. Owing to ethnic intermarriage among Mongolians and Han individuals, the number of patients having pure Mongolian blood decreased significantly. Second, we mainly relied on follow-up visits to observe drug treatment; therefore, there was a great amount of uncertainty. Moreover, some clinical data were lost in the process of follow-up. Additional studies are needed to verify the relationship between *CYP2C19* polymorphisms and clinical treatment strategies.

## Conclusions

In summary, our findings supported the differential distributions of *CYP2C19* genetic polymorphisms and metabolic phenotypes between Mongolian and Han individuals. Moreover, we provided a theoretical basis for drug management and prediction of clinical prognosis according to individual genotypic variations. Ultimately, these findings provided insights into the development of individualized drug therapies among ethnic minority populations in China.
